# Climate Change and Local Public Health in the United States: Preparedness, Programs and Perceptions of Local Public Health Department Directors

**DOI:** 10.1371/journal.pone.0002838

**Published:** 2008-07-30

**Authors:** Edward W. Maibach, Amy Chadwick, Dennis McBride, Michelle Chuk, Kristie L. Ebi, John Balbus

**Affiliations:** 1 Center for Climate Change Communication, George Mason University, Fairfax, Virginia, United States of America; 2 The Pennsylvania State University, State College, Pennsylvania, United States of America; 3 Milford Department of Health, Milford, Connecticut, United States of America; 4 National Association of County & City Health Officials, Washington D. C., United States of America; 5 ESS, LLC, Alexandria, Virginia, United States of America; 6 Environmental Defense, Washington D. C., United States of America; University of Liverpool, United Kingdom

## Abstract

While climate change is inherently a global problem, its public health impacts will be experienced most acutely at the local and regional level, with some jurisdictions likely to be more burdened than others. The public health infrastructure in the U.S. is organized largely as an interlocking set of public agencies at the federal, state and local level, with lead responsibility for each city or county often residing at the local level. To understand how directors of local public health departments view and are responding to climate change as a public health issue, we conducted a telephone survey with 133 randomly selected local health department directors, representing a 61% response rate. A majority of respondents perceived climate change to be a problem in their jurisdiction, a problem they viewed as likely to become more common or severe over the next 20 years. Only a small minority of respondents, however, had yet made climate change adaptation or prevention a top priority for their health department. This discrepancy between problem recognition and programmatic responses may be due, in part, to several factors: most respondents felt personnel in their health department–and other key stakeholders in their community–had a lack of knowledge about climate change; relatively few respondents felt their own health department, their state health department, or the Centers for Disease Control and Prevention had the necessary expertise to help them create an effective mitigation or adaptation plan for their jurisdiction; and most respondents felt that their health department needed additional funding, staff and staff training to respond effectively to climate change. These data make clear that climate change adaptation and prevention are not currently major activities at most health departments, and that most, if not all, local health departments will require assistance in making this transition. We conclude by making the case that, through their words and actions, local health departments and their staff can and should play a role in alerting members of their community about the prospect of public health impacts from climate change in their jurisdiction.

## Introduction

*“Climate change is one of the most serious public health threats facing our nation. Yet few Americans are aware of the very real consequences of climate change on the health of our communities, our families and our children.”* [1]

Georges Benjamin, MD, Executive Director

American Public Health Association

*“We now face a new and unprecedented change: climate change. (It is) perhaps the greatest environmental health challenge for the remainder of our careers, and perhaps for all those (public health professionals) who will follow us.”* [2]

Howard Frumkin, MD, Dr.PH, Director,

National Center for Environmental Health, CDC

*“We the undersigned believe that climate change is the public health challenge of the 21^st^ Century and that, unless decisive action is taken now, the world will face global public health and environmental catastrophe.”* [3]

Alan Maryon-Davis, President

Faculty of Public Health

(and 20 other CEO-level co-signers of British health & sustainability organizations)

*“We need to… convince the world that humanity really is the most important species endangered by climate change.”* [4]

Margaret Chan, MD, Director-General

World Health Organization

The current and potential future toll of climate change on human health is becoming increasingly clear [5]–[7]. Earth system changes associated with climate change–rising temperatures, increasing climate variability, increasing rainfall in some areas and drought in others, more frequent severe weather events, rising sea levels–have both *direct* potential to harm human health through increased heat stress, traumatic injuries and mental health consequences of climate-related disasters, and *indirect* potential through changes in air pollution and aeroallergens, infectious diseases, and ultimately the likelihood of large-scale population dislocation, and civil conflict [6], [8]–[12]. Climate change-induced alterations of ecosystems–i.e., patterns of pests, parasites and pathogens affecting wildlife, livestock, agriculture, forests and coastal marine organisms–can also have negative implications for human health [13].

Over the past year, a growing number public health leaders in the United States and internationally have issued strong statements defining climate change as a critical threat to the public's health (see examples above). Efforts are currently underway to ensure that public health professionals–as well as public officials who oversee public health infrastructure–are aware of and understand the threat of climate change to human health. For example, World Health Day 2008 (April 7^th^) was themed *Protecting Health from Climate Change*, and National Public Health Week, 2008 in the U.S. (April 7th–13th) was themed *Climate Change: Our Health in the Balance*. The medical community is also becoming active on the issue. Leading medical journals, for example Lancet [14]–[19] and British Medical Journal [20], have recently released theme issues focused on climate change and health.

While climate change is inherently a global problem, the public health impacts of climate change will mostly be experienced at the local level, and some regions will be more burdened than others. The public health infrastructure in the U.S. is organized largely as an interlocking set of public agencies at the federal, state and local level, with lead responsibility for each city or county often residing at the local level. Thus, there is a critical need to understand the current knowledge and perceptions of local public health officials regarding public health impacts of climate change and assess current preparedness for these impacts in the U.S. For this reason, we conducted a nationally representative survey of local health department directors.

With this research, we sought to answer four primary questions:

RQ 1: What are local health department director's perceptions of climate change and its potential public health effects?
RQ 2: How prepared are local health departments to address potential health impacts of climate change?
RQ 3: What activities are local health departments currently performing, or planning, that can help prevent further climate change?
RQ 4: What resources do local health departments need to better address climate change?

## Methods

The 2,296 members of the National Association of County & City Health Officials (NACCHO) provided the sampling frame for this survey. A quota sample–with 12 strata based on four regions of the country (defined as U.S. Census regions) and 3 jurisdictional population sizes (small defined as less than 50,000, medium defined as 50,000 to 499,999, and large defined as 500,000 and higher)–was randomly drawn from the universe of possible respondents. Using a method previously designed and used by NACCHO, to determine the size of each stratum we “split the difference” between assigning an equal number of possible respondents to each stratum and assigning a number proportional to their representation in the universe of NACCHO members. A total of 250 NACCHO members were initially drawn for the sample. Of those, 33 were removed from the list because they: were duplicate names (i.e., when one person was the Director or Health Officer for several small jurisdictions; n = 11); represented public health nursing services (n = 6), home care services (n = 2), or boards of health (n = 7); had inaccurate contact information for which no correct information could be found (n = 6); were an investigator on this project (n = 1). Thus, the final sample size was 217.

We developed a telephone interview instrument to measure key constructs associated with our research questions. To measure perceptions of climate change and its potential public health effects on the jurisdiction, we asked four 4-point Likert-type questions (i.e., strongly disagree, disagree, agree, strongly agree, with an option to respond “don't know;” see Table 1 for wording of the questions). Also, for each of 12 specific threats to health that are potentially caused or exacerbated by climate change (see Table 2 for a listing of the specific items) we asked two questions: (1) “Has climate change already affected [this health problem] in your jurisdiction?”; and (2) “Over the next 20 years will climate change make [this problem] more common or severe, less common or severe, or will it remain the same in your jurisdiction over the next 20 years?” Lastly, to assess the relative priority of addressing climate change (as compared to other public health priorities), we asked: “Would you say that preventing or preparing for the public health consequences of climate change is among your health department's top 10 current priorities?” Respondents who indicated that climate change was a top 10 priority were also asked to specify which number–1 to 10, with 1 being the highest priority–best characterized the current priority being accorded climate change in their health department.

**Table 1:** Local health department director's perceptions about general climate change impacts and its priority.

Statement	SD	D	A	SA	DK
My jurisdiction has experienced climate change in the past 20 years.	0.8 (1)	10.5 (14)	60.2 (80)	9.0 (12)	19.5 (26)
My jurisdiction will experience climate change in the next 20 years.	0.8 (1)	2.3 (3)	55.6 (74)	22.6 (30)	18.8 (25)
In the next 20 years, it is likely that my jurisdiction will experience one or more serious public health problems as a result of climate change.	1.5 (2)	8.3 (11)	48.1 (64)	11.3 (15)	30.8 (41)
Preparing to deal with the public health effects of climate change is an important priority for my health department.	3.8 (5)	40.6 (54)	39.1 (52)	12.0 (16)	4.5 (6)

The first entry in each cell is the percent of respondents; second is the actual number of respondents. SD = Strongly Disagree; D = Disagree; A = Agree; SA = Strongly Agree; DK = Don't Know.

**Table 2:** Local health department director's perceptions about specific local impacts of climate change.

	Has climate change already affected this health issue in your jurisdiction?				Over the next 20 years, will climate change make this issue *more* common or severe, *less* common or severe, or will it remain the *same* in your jurisdiction?				
Health issue:	Yes	No	DK	NA	More	Less	Same	DK	NA
Heat waves and heat-related illnesses	56.4 (75)	33.1 (44)	8.3 (11)	2.3 (3)	72.9 (97)	0.8 (1)	15.0 (20)	8.3 (11)	3.0 (4)
Storms (including hurricanes) and floods	47.4 (63)	41.4 (55)	11.3 (15)	0.0 (0)	57.9 (77)	1.5 (2)	24.1 (32)	16.5 (22)	0.0 (0)
Droughts, forest fires, or brush fires	46.6 (62)	40.6 (54)	9.0 (12)	3.8 (5)	59.4 (79)	0.8 (1)	19.5 (26)	18.0 (24)	2.3 (3)
Vector-borne infectious diseases	42.1 (56)	38.3 (51)	19.5 (26)	0.0 (0)	56.4 (75)	3.0 (4)	21.1 (28)	19.5 (26)	0.0 (0)
Water- and food-borne diseases	18.0 (24)	64.7 (86)	16.5 (22)	.08 (1)	36.1 (48)	1.5 (2)	34.6 (46)	27.1 (36)	0.8 (1)
Anxiety, depression or other mental health conditions	21.1 (28)	45.9 (61)	27.8 (37)	5.3 (7)	40.6 (54)	0.8 (1)	19.5 (26)	36.1 (48)	3.0 (4)
Quality or quantity of fresh water available to your jurisdiction	42.9 (57)	40.6 (54)	12.8 (17)	3.8 (5)	63.2 (84)	3.0 (4)	18.0 (24)	13.5 (18)	2.3 (3)
Quality of the air, including air pollution, in your jurisdiction	41.4 (55)	37.6 (50)	16.5 (22)	4.5 (6)	65.4 (87)	2.3 (3)	13.5 (18)	15.8 (21)	3.0 (4)
Unsafe or ineffective sewage and septic system operation	12.8 (17)	72.2 (96)	12.8 (17)	2.3 (3)	18.8 (25)	6.0 (8)	47.4 (63)	26.3 (35)	1.5 (2)
Food safety and security	14.3 (19)	74.4 (99)	8.3 (11)	3.0 (4)	30.8 (41)	3.0 (4)	48.9 (65)	15.8 (21)	1.5 (2)
Housing for residents displaced by extreme weather events	18.6 (25)	69.9 (93)	7.5 (10)	3.8 (5)	42.1 (56)	0.0 (0)	37.6 (50)	18.0 (24)	2.3 (3)
Health care services for people with chronic conditions during service disruptions, such as extreme weather events	25.6 (34)	59.4 (79)	11.3 (15)	3.8 (5)	53.4 (71)	1.5 (2)	30.1 (40)	12.0 (16)	3.0 (4)

The first entry in each cell is the percent of respondents; second is the actual number of respondents.

DK = Don't Know; NA = No answer was given.

To operationalize preparedness, we measured: (1) perceptions of how knowledgeable key stakeholders in the jurisdiction are about climate change (using 6 Likert-type items; see Table 3); (2) perceptions of expertise on climate change mitigation and adaptation planning available to the health department (using 7 Likert-type items; see Table 4); (3) whether the health department was currently operating a program to address each of the 12 specific threats to health; (4) whether the health department was currently or planning to incorporate climate change adaptation into the planning for each program that they were operating (see Table 5); and (5) whether the health department was currently using long-range weather or climate information in planning or implementing each of the programs that they were operating.

**Table 3:** Local health department director's perceptions of climate change knowledge in their health department and among other relevant leaders in the jurisdiction.

Statement	SD	D	A	SA	DK	NA
I am knowledgeable about the potential public health impacts of climate change	2.3 (3)	28.6 (38)	60.9 (81)	4.5 (6)	3.0 (4)	0.8 (1)
The other relevant senior managers in my health department are knowledgeable about the potential public health impacts of climate change	5.3 (7)	36.1 (48)	41.4 (55)	3.8 (5)	12.0 (16)	1.5 (2)
Many of the other relevant appointed officials in my jurisdiction–such as environmental, agricultural, forestry and wildlife, energy, and transportation officials–are knowledgeable about the potential public health impacts of climate change	8.3 (11)	33.1 (44)	27.8 (37)	2.3 (3)	27.8 (37)	0.8 (1)
Many of the relevant elected officials in my jurisdiction are knowledgeable about the potential public health impacts of climate change	16.5 (22)	43.6 (58)	21.8 (29)	0.8 (1)	17.3 (23)	0.0 (0)
Many of the business leaders in my jurisdiction are knowledgeable about the potential public health impacts of climate change	12.0 (16)	45.1 (60)	9.0 (12)	0.8 (1)	33.1 (44)	0.0 (0)
Many of the leaders of the health care delivery system in my jurisdiction–including hospitals and medical groups–are knowledgeable about the potential public health impacts of climate change	2.3 (3)	40.6 (54)	28.6 (38)	2.3 (3)	25.6 (34)	0.8 (1)

The first entry in each cell is the percent of respondents; second is the actual number of respondents.

SD = Strongly Disagree; D = Disagree; A = Agree; SA = Strongly Agree; DK = Don't Know;

NA = No answer was given

**Table 4:** Local health department director's perceptions of expertise available to them on the public health aspects of climate change

Statement	SD	D	A	SA	DK	NA
My health department currently has ample expertise to assess the potential public health impacts associated with climate change that could occur in my jurisdiction.	27.8 (37)	49.6 (66)	18.8 (25)	3.8 (5)	0.0 (0)	0.0 (0)
My health department currently has ample expertise to create an effective climate change *adaptation* plan.	31.6 (42)	51.1 (68)	15.8 (21)	0.8 (1)	0.0 (0)	0.8 (1)
My state health department currently has ample expertise to help us create an effective climate change *adaptation* plan in this jurisdiction.	18.8 (25)	34.6 (46)	22.6 (30)	3.0 (4)	21.1 (28)	0.0 (0)
The Centers for Disease Control and Prevention currently has ample expertise to help us create an effective climate change *adaptation* plan in this jurisdiction.	1.5 (2)	16.5 (22)	31.6 (42)	2.3 (3)	48.1 (64)	0.0 (0)
The health care delivery system in my jurisdiction–including hospital and medical groups–has ample expertise to help us create an effective climate change *adaptation* plan.	22.6 (30)	41.4 (55)	15.8 (21)	0.8 (1)	18.8 (25)	0.8 (1)
My health department currently has ample expertise to create an effective climate change *mitigation* plan.	38.3 (51)	47.4 (63)	11.3 (15)	0.0 (0)	1.5 (2)	1.5 (2)
My state's health department currently has ample expertise to help us create an effective climate change *mitigation* plan in this jurisdiction.	21.8 (29)	37.6 (50)	15.8 (21)	0.0 (0)	24.8 (33)	0.0 (0)
The Centers for Disease Control and Prevention currently has ample expertise to help us create an effective climate change *mitigation* plan in this jurisdiction.	6.0 (8)	18.0 (24)	21.8 (29)	3.0 (4)	51.1 (68)	0.0 (0)

The first entry in each cell is the percent of respondents; second is the actual number of respondents.

SD = Strongly Disagree; D = Disagree; A = Agree; SA = Strongly Agree; DK = Don't Know;

NA = No answer was given.

**Table 5:** Climate change adaptation activities of local health departments

	Is this a current activity in your health department?			Do you *currently* or are you *planning* to incorporate climate change adaptation into your planning?			
Health issue:	Yes	No	DK	Currently	Planning	No	DK
Heat waves and heat-related illnesses	57.1 (76)	42.1 (56)	0.8 (1)	21.8 (29)	17.3 (23)	58.6 (78)	2.3 (3)
Storms (including hurricanes) and floods	76.7 (102)	23.3 (31)	0.0 (0)	36.1 (48)	20.3 (27)	40.6 (54)	3.0 (4)
Droughts, forest fires, or brush fires	37.6 (50)	62.4 (83)	0.0 (0)	13.5 (18)	10.5 (14)	72.2 (96)	3.8 (5)
Vector-borne infectious diseases	94.7 (126)	4.5 (6)	0.8 (1)	39.8 (53)	13.5 (18)	43.6 (58)	3.0 (4)
Water- and food-borne diseases	97.0 (129)	2.3 (3)	0.8 (1)	35.3 (47)	14.3 (19)	45.9 (61)	3.8 (5)*
Anxiety, depression or other mental health conditions	30.8 (41)	68.4 (91)	0.8 (1)	7.5 (10)	7.5 (10)	80.5 (107)	4.5 (6)
Quality or quantity of fresh water available to your jurisdiction	66.9 (89)	33.1 (44)	0.0 (0)	16.5 (22)	19.5 (26)	57.9 (77)	6.0 (8)
Quality of the air, including air pollution, in your jurisdiction	50.4 (67)	48.9 (65)	0.8 (1)	20.3 (27)	12.0 (16)	64.7 (86)	3.0 (4)
Unsafe or ineffective sewage and septic system operation	78.9 (105)	20.3 (27)	0.8 (1)	30.1 (40)	7.5 (10)	57.9 (77)	4.5 (6)
Food safety and security	89.5 (119)	10.5 (14)	0.0 (0)	33.1 (44)	13.5 (18)	48.9 (65)	4.5 (6)
Housing for residents displaced by extreme weather events	37.6 (50)	60.2 (80)	1.5 (2)	18.8 (25)	12.8 (17)	64.7 (86)	3.8 (5)
Health care services for people with chronic conditions during service disruptions, such as extreme weather events	57.1 (76)	41.4 (55)	1.5 (2)	26.3 (35)	15.0 (20)	54.9 (73)	3.8 (5)
Emergency preparedness for the above issues				56.4 (75)	14.3 (19)	25.6 (34)	3.8 (5)

The first entry in each cell is the percent of respondents; second is the actual number of respondents. DK = Don't Know.

* One respondent did not answer this question.

To measure mitigation (i.e., primary prevention) activities, we asked if the health department currently had, or was planning to have, a program focused on each of 8 specific objectives (see Table 6). And lastly, to measure perceived resource needs, we asked the following open-ended question: “Are there resources that your department does not currently have that, if made available, would significantly improve its ability to deal with climate change as a public health issue?” If respondents answered “yes,” follow-up questions were asked to determine the nature of those resources.

**Table 6:** Climate change mitigation activities of local health departments

	Do you *currently* have or are you *planning* to have a program focused on this activity?			
A program:	Currently	Planning	No	DK
A program focused on mitigating climate change by reducing greenhouse gas emissions from the health department?	12.0 (16)	14.3 (19)	68.4 (91)	5.3 (7)
A program to help residents of your jurisdiction reduce their greenhouse gas emissions?	5.3 (7)	7.5 (10)	83.5 (111)	3.8 (5)
A program to reduce fossil fuel use or conserve energy in the operation of the health department?	21.1 (28)	18.8 (25)	54.9 (73)	5.3 (7)
A program to help residents of your jurisdiction reduce their fossil fuel use or conserve energy?	6.0 (8)	8.3 (11)	80.5 (107)	5.3 (7)
A program to encourage or help people to use active transportation such as walking, cycling?	50.4 (67)	11.3 (15)	36.1 (48)	2.3 (3)
A program to encourage or help people to use mass transportation?	15.0 (20)	6.0 (8)	76.7 (102)	2.3 (3)
A program to encourage or help people to change the way they purchase foods such as buying locally-grown foods, organic foods, or plant-based foods?	33.8 (45)	9.0 (12)	54.1 (72)	3.0 (4)
A program to educate the public about climate change and its potential impact on health?	8.3 (11)	18.0 (24)	69.9 (93)	3.8 (5)

The first entry in each cell is the percent of respondents; second is the actual number of respondents. DK = Don't Know.

One investigator (AC) pre-tested the instrument for length, clarity and comprehension with a convenience sample of six local health department directors who were recruited at the 2007 NACCHO Annual Meeting. Pre-test interviews took approximately 45 minutes to complete. After completing the interview, participants were asked to comment on the survey and the administration format. Minor revisions were made and the instrument was finalized.

Twelve trained interviewers (including authors AC and MC) conducted the interviews. The interviewers represented all of the investigators' institutions. Using an interviewer manual developed to support interviewer training, one investigator (AC) trained all the interviewers. The training consisted of general information about conducting interviews, a review of the study's protocol, question-by-question review of the survey, and practice sessions for recruitment and survey scripts. The participants were trained in two sessions that lasted four hours total with additional time for practicing the scripts with a partner. An investigator (AC) reviewed each interviewer's initial interview before she or he conducted additional interviews. The survey instrument and the interviewer manual are available upon request.

On November 2, 2007, members of the sample were e-mailed a letter from NACCHO's Executive Director and Senior Advisor for Environmental Health (MC) that described the purpose of the survey and encouraged members to participate in the survey when an interviewer called them. Approximately one week later, interviewers began contacting participants via e-mail and telephone to request an interview. Approximately five contact attempts were made to schedule an interview before a participant was considered a passive refusal. Most of the interviews (79%, n = 105) were completed by December 22, 2007. In mid-January, a smaller set of interviewers attempted to schedule interviews with participants who were previously too busy to participate. The fielding of the survey ended February 22, 2008.

A total of 133 members of the sample agreed to be interviewed and completed the survey. Of the remaining members of the sample, 18% (n = 38) actively refused to participate, and 21% (n = 46) passively refused by virtue of not responding to interviewer calls or emails. Thus, the response rate and survey completion rate for this study was 61%.

All data were entered into Excel, with verification. For this article, only the quantitative data were analyzed. These data were imported into SPSS version14.0 for analysis.

## Results

### Research Question 1: What are local health department directors' perceptions of climate change and its potential public health effects?

The majority of local health department directors surveyed perceived climate change to be a relevant threat in their jurisdiction (see Table 1). Nearly 70 percent believed that their jurisdiction had experienced climate change in the past 20 years, and 78 percent believed their jurisdiction would experience climate change in the next 20 years. Approximately 60 percent believed their jurisdiction would experience one or more serious public health problems as a result of climate change over the next 20 years, while fewer than 10 percent believed their jurisdiction would not experience such problems.

A significant proportion of respondents believed that climate change had already affected 12 distinct threats to health in their jurisdiction (e.g., vector-borne infectious diseases; see Table 2). Participants were least likely to believe that safety of the sewage or septic systems (13%) and food safety and security (14%) had already been affected by climate change in their jurisdiction, and were most likely to believe that heat waves and heat-related illness (56%) and storms and floods (47%) had already been affected by climate change. Most respondents felt that, as a result of climate change, at least some these threats would become more common or severe in their jurisdiction over the next 20 years. Specifically, they believed that heat waves and heat-related illness (73%), reduced air quality (65%), reduced water quality or quantity (63%), and droughts, forest fires and brush fires (59%) were most likely to become more common or severe as a result of climate change.

Despite their recognition of climate change as a threat to health in their jurisdiction, relatively few respondents reported that climate change was a top priority for their health department. While about half of our respondents “agreed” or “strongly agreed” that preparing to deal with the public health effects of climate change was a priority for their health department, relatively few of them strongly agreed (see Table 1). Moreover, in response to a follow-up question, only 19 percent of respondents indicated that climate change was among their department's top 10 current priorities, and only 6 percent indicated climate change was one of their health department's current top five priorities.

### Research Question 2: How prepared are local heath department to address potential health impacts of climate change?

#### Perceived Knowledge

Most respondents (approximately two-thirds) felt that they themselves were knowledgeable about the potential public health impacts of climate change, but fewer than half felt that other relevant senior managers in their health department were similarly knowledgeable (see Table 2). Moreover, less than one third of respondents felt that other relevant stakeholders in their community (i.e., appointed and elected officials, business leaders, and health care delivery leaders) had knowledge of the potential public health impacts of climate change (see Table 3). It is important to note that very few respondents (less than 5%) “strongly agreed” that any key stakeholder group in their community, including themselves, was knowledgeable about the issue.

#### Perceived Expertise

The large majority of directors (77%) believed that their health department lacked expertise in assessing the public health risks of climate change in their jurisdiction, and lacked expertise to create either an effective adaptation plan (83%) or an effective mitigation plan (86%; see Table 4). Relatively few respondents believed that their state health department currently had ample expertise to help them create an adaptation plan (26%) or a mitigation plan (16%). Similarly, relatively few respondents believed that the Centers for Disease Control and Prevention (CDC) currently had ample expertise to help them create an adaptation plan (34%) or a mitigation plan (25%) for their jurisdiction.

#### Current Programs of the Health Department

Nearly all respondents indicated that their health department currently had program activity that addresses at least some of the 12 potential direct effects of climate change on the public's health (see Table 5). The most common areas of relevant programmatic activity were water- and food-borne diseases (97%), vector-borne infectious diseases (95%) and food safety and security (90%). The least common areas of programmatic activity were anxiety, depression and mental health conditions (31%), droughts, forest fires and brush fires (38%) and housing for residents displaced by extreme weather events (38%).

#### Climate Change Adaptation Programs of the Health Department

Some respondents indicated that they currently, or plan to, incorporate climate change adaptation into at least some of their programmatic activities (see Table 5). The most common areas of current or future programmatic activity related to climate change were emergency preparedness (71%), storms and floods (56%), vector-borne infectious diseases (53%), and water- and food-borne diseases (50%). The least common were anxiety, depression and other mental health conditions (15%), droughts, forest fires and brush fires (24%), housing for residents displaced by extreme weather events (32%), and air quality (32%).

#### Use of Long-Range Weather or Climate Information

Only 29% of respondents indicated that their health department currently uses long-range weather or climate information in planning or operating any of their programs. Among those departments using such information, the average number of programs in which the information is used was 5.5

### Research Question 3: What activities are local health departments currently performing, or planning, that can help prevent further climate change?

Although climate change mitigation per se appears to be an area of current activity for relatively few local health departments, a substantial proportion of health departments do have programs in areas consistent with mitigation objectives (see Table 6). The most common relevant current programs are those that encourage active transportation such as cycling and walking (50%) and programs that encourage purchase of local grown, organic or plant-based foods (34%). The least common are those than pertain directly to climate change mitigation, including programs to help residents reduce their greenhouse gas emissions (5%), programs to reduce residents' fossil fuel use or conserve energy (6%), and programs to educate the public about the potential impact of climate change on health (8%).

Relatively few health departments are currently planning new public programs directly or indirectly relevant to mitigation. The most common of these were public education programs about the potential impact of climate change on health (17%) and active transportation programs (11%). The least common were programs to encourage use of mass transportation (6%) and programs to help residents reduce their greenhouse gas emissions (8%) or fossil fuel use (8%).

Of special note are current and planned efforts by health departments to reduce the greenhouse gas emissions and energy use associated with operation of their health department. Relatively few health departments currently have a program to reduce fossil fuel use or conserve energy in health department operations (21%) or to specifically reduce their greenhouse gas emissions (12%), and relatively few others are planning such programs (19% and 14%, respectively).

### Research Question 4: What resources do local health departments need to better address climate change?

The large majority of respondents (77%) indicated that additional resources, if available, would significantly improve their department's ability to deal with climate change as a public health issue. A small segment of respondents (9%) indicated that additional resources were not needed, and another small segment (14%) indicated that they did not know if additional resources would be helpful or not.

Among respondents who indicated that additional resources would be helpful, the categories of resources specified were the following: additional funding to support the activity (63%), additional staff (54%), staff training (29%), equipment (10%), and assorted other resources (17%).

## Discussion

This is the first nationally representative survey to assess the perceptions and activities of local public health directors regarding climate change and public health. As such, it provides a valuable baseline for the public health community as it increases the intensity of its efforts to respond to climate change. Overall, our survey points to relatively widespread awareness of the importance of climate change for public health among directors of local health departments, but far lower levels of actual preparedness or planned activities to detect, prevent and ameliorate climate-associated health problems. These findings extend, and are largely consistent with, a recently released study of local public health department directors in California [21].

A majority of the local health department directors who responded to our survey felt that climate change was already a problem in their jurisdiction and is likely to become more of a problem over the next 20 years, yet only a small minority had yet to make climate change one of their department's top priorities. There may be many reasons for this response. The results of our survey suggest that key factors may include lack of knowledge about climate change–both within the local public health sector and among other key stakeholders in the community–and the perceived lack of adaptation and mitigation planning expertise in the public health community at large. Additional factors may include that other public health priorities are seen as being more immediately pressing (e.g., pandemic flu preparedness), and that there is a chronic lack of resources in most local public health departments, a factor that undermines their ability to effectively address any of their top priorities. Respondents to our survey offered additional perspectives in their open-ended comments, but in the interest of bringing our main findings forward as rapidly as possible, that information is not included in this paper; analysis of the open-ended responses will begin shortly.

While addressing the energy, transportation, economic, and environmental implications of climate change has increasingly become a priority for the United States, the health implications of climate change have largely been neglected. Research funding for health impacts of climate change has been a relatively small part of the overall U.S. Climate Change Science Program (CCSP), and a National Academy of Science review committee has called on the CCSP to place greater priority on health impacts [22]. Current legislation regarding climate change generally omits measures to assess and remediate health impacts. By highlighting the strengths and gaps in our public health infrastructure's preparedness for climate-related impacts, this survey can help inform research and legislative efforts to reduce climate change impacts through mitigation and adaptation efforts.

Frumkin and his colleagues (at CDC's National Center for Environmental Health) recently suggested that public health agencies–in coordination with academic institutions, non-governmental organizations, and the private sector–are well positioned to respond to climate change by building on the essential public health services that they already provide [23]. Our data support this position, albeit indirectly, by documenting the significant extent of programmatic activity relevant to climate change adaptation in most local health departments, even if most of this activity does not yet specifically address climate change. A small proportion of local health departments have begun to leverage their resources to protect the public's health from climate change, and others are beginning to consider how they will do so. Our data make clear, however, that climate change adaptation is not currently a major activity at most health departments, and that most, if not all, will require assistance in making this transition.

Our findings also indicate that climate change prevention is not currently a priority in the large majority of local health departments. We see this as both a problem and an opportunity. Most Americans view climate change as a threat to other species (e.g., polar bears) and to elements of the environment (e.g., glaciers), rather than as a threat to people [24]. That may be, in part, because the voice of public health professionals–a highly respected community that has a unique voice in promoting activities that can prevent adverse health impacts–has been nearly silent on the issue of climate change. Public health and health care professionals have myriad opportunities to make the case (in the media, at county or city council meetings, etc.) that climate change is a profound threat to the health and wellbeing of people, and we urge them to do so. Of particular importance for local public health department directors is the need to make the case that climate change threatens the health of people in their jurisdiction. Most people associate climate change primarily as a threat to things distant from them geographically, and temporally, rather than as a direct threat to their community. [24]. This abstraction may impede effective individual responses and appropriate behavior changes.

One additional finding is particularly worthy of note: the lack of focus among local health department directors on reducing greenhouse gas emissions from health department operations. Admittedly, the aggregate contribution of greenhouse gas emissions from local public health departments is inconsequentially small (although the same is not true of the health care delivery sector as a whole, which contributes substantially to the overall level of U.S. emissions). For the reasons articulated above, however, we see this lack of action as a symbolically important missed opportunity. Because of the seriousness of climate change's threat to public health, public health departments should reduce energy use and greenhouse gas emissions to the best of their ability. Such efforts made publicly can reinforce the message that climate change is a threat to human health, and provide a model for appropriate mitigation actions for citizens and other organizations.

A comment about the limitations of our research is also in order. While the response rate to our survey was robust (61%), it is possible that non-respondents differed from respondents in critical ways. We believe that a significant proportion of our non-response rate is attributable to the timing of the survey: the survey was conducted during the year-end holiday season, and again in January during a large national influenza outbreak; anecdotally, at least some people who actively refused to participate cited that they were simply too busy to spend a half hour or more being interviewed. It is also possible, however, that some of the non-participants were more likely than the participants to believe that climate change is not a significant issue for the public health community. Anecdotally, at least several people who actively refused to be interviewed made comments to the effect of the concern about climate change being “blown out of proportion.” Thus, there is a possibility that our findings may be overestimating the true level of awareness and perception of seriousness of climate change impacts among local public health directors nationwide.

The results reported here are descriptive only. Further research is needed to determine what factors predispose and enable local health departments to play an active and effective role in climate change adaptation and mitigation. We continue to analyze the results of this survey for that purpose and encourage others to engage in similar lines of inquiry.
